# Hydrothermal synthesis of zinc oxide nanoparticles using rice as soft biotemplate

**DOI:** 10.1186/1752-153X-7-136

**Published:** 2013-08-06

**Authors:** Donya Ramimoghadam, Mohd Zobir Bin Hussein, Yun Hin Taufiq-Yap

**Affiliations:** 1 Material Synthesis and Characterization Laboratory (MSCL), Institute of Advanced Technology (ITMA), Universiti Putra Malaysia, 43400 UPM, Serdang, Selangor, Malaysia; 2 Research center for Catalysis Science and Technology PutraCAT, Faculty of Science, Universiti Putra Malaysia, 43400 UPM, Serdang, Selangor, Malaysia

**Keywords:** Zinc oxide nanoparticles, Rice, Biotemplate, Soft templating, Starch, Hydrothermal method

## Abstract

**Background:**

Rice as a renewable, abundant bio-resource with unique characteristics can be used as a bio-template to synthesize various functional nanomaterials. Therefore, the effect of uncooked rice flour as bio-template on physico-chemical properties, especially the morphology of zinc oxide nanostructures was investigated in this study.

The ZnO particles were synthesized through hydrothermal-biotemplate method using zinc acetate-sodium hydroxide and uncooked rice flour at various ratios as precursors at 120°C for 18 hours.

**Results:**

The results indicate that rice as a bio-template can be used to modify the shape and size of zinc oxide particles. Different morphologies, namely flake-, flower-, rose-, star- and rod-like structures were obtained with particle size at micro- and nanometer range. Pore size and texture of the resulting zinc oxide particles were found to be template-dependent and the resulting specific surface area enhanced compared to the zinc oxide synthesized without rice under the same conditions. However, optical property particularly the band gap energy is generally quite similar.

**Conclusion:**

Pure zinc oxide crystals were successfully synthesized using rice flour as biotemplate at various ratios of zinc salt to rice. The size- and shape-controlled capability of rice to assemble the ZnO particles can be employed for further useful practical applications.

## Background

Remarkable improvements have been made using bio-inspired approach for materials synthesis due to possible control of physico-chemical properties. A technique that employs natural materials as biotemplates to synthesize micro- and nano-scaled materials with morphologies and structures resemble to those of the biotemplate is called as biomorphic mineralization [1]. These kinds of works keep on growing and contributing to a new interdisciplinary areas, especially with the synthesis, self-assembly and processing of the organized inorganic materials [2].

Biotemplating approach is an impressive strategy to achieve the morphology-controllable materials with structural specialty, complexity and relevant fascinating functions. The advantages of applying biotemplates are that they are relatively cheap, economical, environmentally benign and renewable [3]. A series of natural biotemplates that were utilized in the fabrication of functional materials includes DNA [4], proteins [5], viruses [6], bacteria [7], diatoms [8], pollen grains [9], shell membrane, wood [10] and cellulose fibers [11].

Application of metal oxides materials have extensively arisen throughout human civilization and the uses of nano-sized particles are even more significant. Among them, ZnO nanoparticles are always in the center of attention due to their fascinating properties and extensive application. Bio-inspired synthesis of ZnO nanoparticles has been achieved using environmentally and eco-friendly accepted systems. Several studies have been investigated the use of natural materials for ZnO nanoparticles synthesis such as DNA [12], silk [13], albumen [14], orange juice [15], pea starch [16], peptide structures [17] and etc. The use of nanoparticles derived from noble metals has spread in many areas involving medical fields, electronics, antibacterial textiles, etc. As a matter of fact, orientation, size and physical properties of nanoparticles affect the performance and reproducibility of a potential device. Thus the synthesis and assembly of shape- and size-controlled nanocrystals are essential components for any practical applications.

Agricultural materials particularly those containing cellulose indicate potential metal bio-sorption capacity. The basic components of the agricultural materials include hemi-cellulose, lignin, extractives, lipids, proteins, etc. [2]. Rice is an agricultural bio-resource which can be used as non-metalic bio-precursor to synthesize functional materials [1]. The main component of rice is starch which is one of the most fascinating bioresource that can be used for nanotechnology application. The carbohydrate polymeric chains build up from glucose units and parted in linear amylase and branched amylopectin. These peculiarities are representing the key structural elements for the synthesis of new functional nanomaterials [18]. Starch-based oxides with biocompatible and non-toxic features, grant a new class of functional nanomaterials with potential application in various industries. Therefore using rice as a soft biotemplate appears to be a promising way to synthesize zinc oxide nanoparticles. In our study, rice was chosen to be used as a soft template material due to its high porous structure, special components and relatively low cost. Lots of studies have been conducted to investigate the use of rice husk [1,19] and starches [20,21] for the synthesis of various functional materials. However, to the best of our knowledge, no such study on the synthesis of ZnO nanoparticles using rice as biotemplate is found in the open literature. Therefore, this study will contribute some input to the body of knowledge and worth to be carried out. The main objective of this research is to determine the effects of uncooked rice as a biotemplate on ZnO physico-chemical properties, particularly its morphology and surface properties. Due to easy controlling of the size, shape and water solubility of ZnO, then the biocompatibility and functionality of ZnO nanomaterials could be further improved by surface modification [13].

## Results and discussion

### XRD analysis

**Figure 1:**
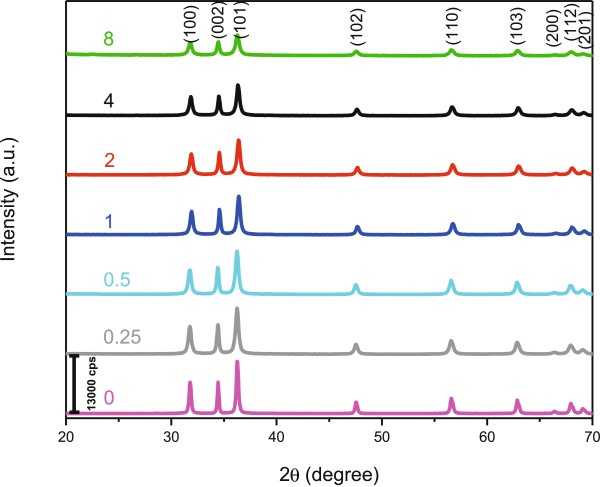
Powder X-ray diffraction (PXRD) patterns of as-synthesized ZnO prepared at different concentrations of uncooked rice powder; 0, 0.25, 0.5, 1, 2, 4, 8 g (w/w%).

Figure 1 shows the XRD patterns of the samples synthesized using different concentrations of uncooked rice powder by hydrothermal method. All the diffraction peaks can be indexed as hexagonal wurtzite-structure (JCPDS card No. 36–1451). The sharp and narrow peaks also illustrate that ZnO particles enjoy high crystallinity and purity. As clearly seen from Figure 1, the intensity of the XRD peaks for as-synthesized ZnO samples was decreased by increasing the concentration of UR, this is due to presence of biotemplate. Even though very little amount of biotemplate present in the sample, no characteristic peaks of rice components can be observed in the XRD pattern. Therefore, employing UR as biotemplate could result in the synthesis of relatively pure ZnO particles.

### Morphology and size

**Figure 2:**
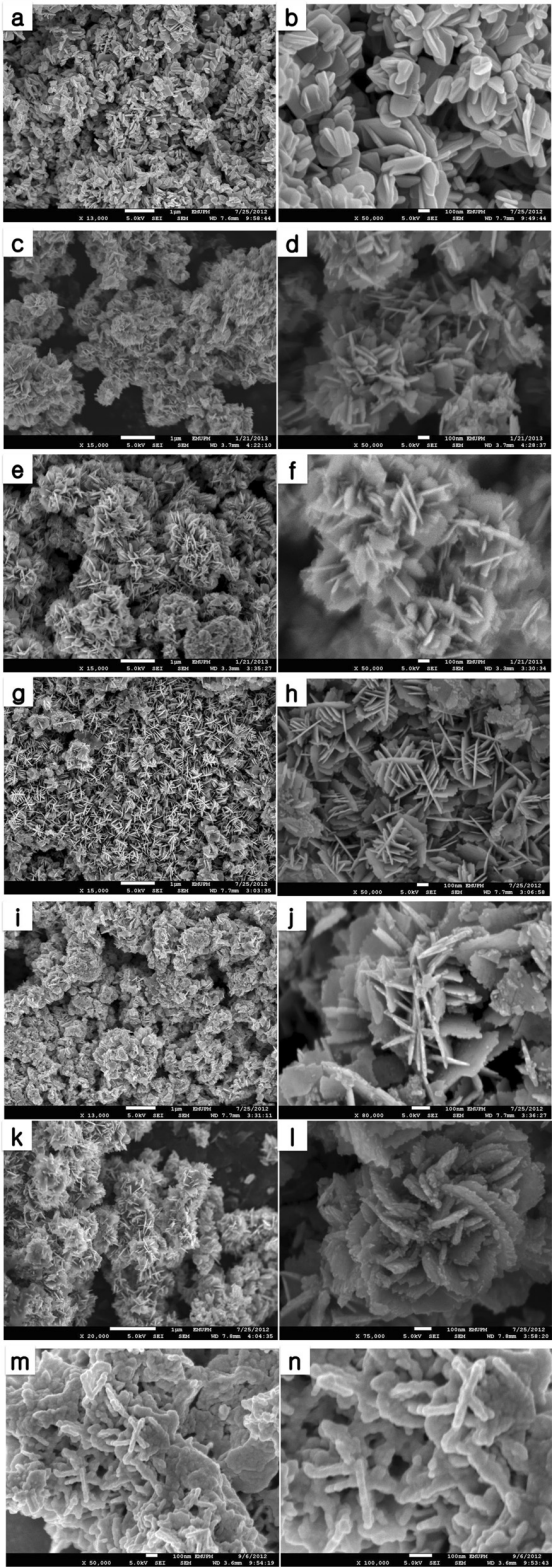
FESEM images of ZnO prepared using different concentrations of uncooked rice (g); 0 (a, b), 0.25 (c, d), 0.5 (e, f), 1 (g, h), 2 (i, j), 4 (k, l), 8 (m, n).

Figure 2 shows field emission scanning electron microscopy (FESEM) images of the samples synthesized at different concentrations of uncooked rice (UR). To investigate the effects of raw rice on the resulting ZnO morphology, FESEM images of ZnO synthesized without UR are also shown in Figure 2a and b. As seen in Figure 2c and d, the ZnO structures show mostly flake-like structures assembling together. They are much more ordered in contrast to the one synthesized without UR (as a control) (Figure 2a and b). The diameter of ZnO flakes dramatically decreased after adding 0.25 g UR. This may occur due to the inhibition of lateral growth of ZnO crystals. Indeed, the accessibility of the zinc ions to the ZnO crystal seeds was controlled by biotemplate. However, size of particles seems to be increased when the synthesis was done using 0.25 g UR. Different morphologies of as-synthesized ZnO was observed with increasing the amount of uncooked rice to 0.5 g. Particles with very small flower-like shape can be observed in Figure 2e and f. Lower magnification of FESEM image indicates that the mentioned structure shows denticulated petals aggregated and form larger flowers of particles. It is notable that the size of the ZnO particles has been obviously decreased for sample prepared using 0.5 g UR. In addition, the tooth-like flakes are more dominant for the ZnO sample prepared using 0.5 g UR compared to the one synthesized using 0.25 g UR.

Figure 2g and h indicate the FESEM images of ZnO sample synthesized using 1 g UR. Very unique star-like structure can be clearly observed at low to high magnification. The star-like structure contains small flakes with denticulated edges which attach to other similar flakes in the center. A closer look shows that the lateral flake act as a substrate for other flakes to grow on the surface and form star-like structure. Similar structure was also reported in literature, confirming that the branched pattern for soft templates, starch, revealing that the semicrystalline granules of starch are made from concentric rings in which the amylose and amylopectin, basic components are aligned perpendiculary to the growth rings and to the granule surface [22]. Figure 2g and h shows that the size of the star-like ZnO particles decreased in comparison with the previous lower amount of uncooked rice.

In the case of ZnO crystals synthesized at 2 g UR, increasing the amount of biotemplate resulted in different morphologies of ZnO particles produced. It formed lots of agglomerated toothed-edge flakes which become a secondary unit for bigger particles. The star-like shape of the particles can be perceived in some areas but aggregation seems to be dominant and prevented clearer observation of the particles as they really are.

Figure 2k and l show FESEM images for as-synthesized ZnO particles synthesized using 4 g UR. The ZnO morphology changed to flower-like structures, mostly rose-like shapes. Detailed view on flower-like particles reveals that their flakes have largest diameter compared to other samples. In the case of ZnO synthesized using 8 g UR, a new morphology, different from other and control samples was observed. The ZnO crystals show mostly rods with around 100 nm size. Moreover, agglomerated without any specific shape particles coexisted with nanorods in the structure of ZnO synthesized using 8 g UR.

The rice main component is carbohydrates which is polysaccharides. In synthesis of nanosized oxide-based materials, carbohydrate can play multiple roles, namely coating/capping, functionalizing, stabilizing, poring and/or coordinating agent. In one hand, polymeric structure of starch with helical-shaped carbonaceous matrix which carrying multiple polyol groups create a protective and functionalized surrounding shield for metal ions which plays a structure-directing role. The hydroxyl groups of amylopectin could be involved both in intra- and/or intermolecular supramolecular association. They are able to coordinate transition metal ions, maintaining the nanoparticles highly aggregated [18,23]. Rice granules swell in aqueous solution and their semi-crystalline structure is lost as the smaller amylose molecules start leaching out of the granule. The small amylose molecules can form complexes with Zn^2+^ because of their high number of coordinating functional groups. It is likely that the majority of the zinc ions are closely associated with the starch molecules, so nucleation and initial crystal growth might preferentially occur within regions of both high starch concentration and high Zn^2+^ concentration [21]. The Van der Waals interactions between the surface molecules of the nanocrystallites form the driving force for self-assembly, and then ZnO nanocrystals can be assembled to form larger ZnO crystals [24], which explain the basis of the growth mechanism of zinc oxide crystals when raw rice was used as template.

**Figure 3:**
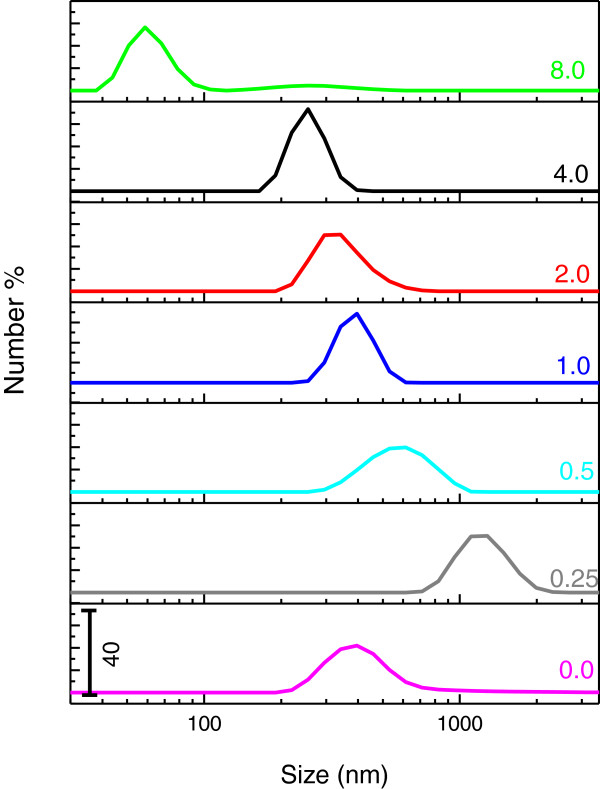
Particle size distribution of ZnO samples synthesized using various concentrations of UR (g); 0, 0.25, 0.5, 1, 2, 4, 8 (w/w%).

Figure 3 shows the particle size distribution of the ZnO samples synthesized using 0.25, 0.5, 1, 2, 4, 8 g UR. Particle size distribution of ZnO synthesized without rice is also given for comparison. As shown in Figure 3, the range of particle size for ZnO synthesized without UR lies between 200–800 nm. When 0.25 g UR was used in the synthesis, size of particles increased dramatically to 800–2000 nm. It is notable that size of ZnO synthesized using 0.5 g UR, considerably decreased to 200–1000 nm range. The decreasing trend continues for sample synthesized at 1 g UR and with a size range of 250–700 nm. Although this distribution is quite similar to that of ZnO synthesized without biotemplate, it is slightly narrower. On the basis of particle size distribution for the samples synthesized using 2 and 4 g UR, it can be clearly observed that the size of particles decreased to 200–700 nm and 150–700 nm, respectively. In the case of ZnO sample synthesized at 8 g UR, the size of particles is at the nano size regime, between 40–100 nm. As mentioned in growth mechanism, adding biotemplate presumably acted as flocculants and forces aggregation. Therefore, the surface-active sites of the template might influence the size and state of aggregation during the particle growth process [25] and finally on the resulting ZnO particles size distribution.

### FTIR spectroscopy

**Figure 4:**
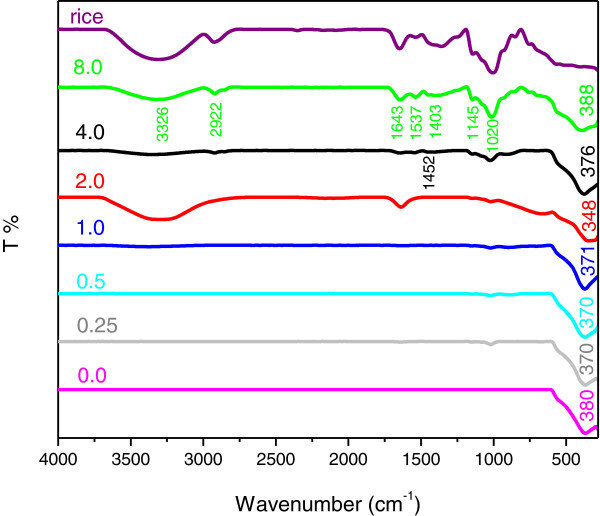
**FTIR spectra of ZnO samples synthesized using various concentrations of UR in the range of 4000–280 cm**^**-1**^** without calcination.**

FTIR spectroscopy was used to investigate the effect of biotemplate (rice) on the resulting chemical properties of the ZnO nanostructures prepared by hydrothermal method. FTIR spectra were obtained at room temperature in the range of 4000–280 cm^−1^. Figure 4 shows the FTIR spectra for the as-prepared ZnO nanostructures synthesized using different concentrations of UR. Generally, rice contains mainly starch (carbohydrates) and water with minor components such as proteins, vitamins, lipids, etc. These compositions mostly consist of alkene, esters, aromatics and alcohols with different combination of functional groups. As shown in Figure 4, the FTIR spectra of the products involve characteristic peaks of rice, such as a broad band at 3300–3400 cm^-1^ is attributed to –OH stretching vibrations, possibly including H_2_O, alcoholic OH, phenolic OH and/or carboxylic OH [26]. A band at 2922 cm^-1^ is assigned to stretching vibrations of aliphatic CH. Moreover, absorption band at 1640 cm^-1^ represents the hydroxyl group of chemisorbed and/or physisorbed H_2_O molecules on the particle surface [27]. Bands at around 1450 and 1400 cm^-1^ are attributed to the C-H bending and angular deformation of C-H bond in starch molecule, respectively [28]. Absorption band at 1145 cm^-1^ ascribed to C-O bond stretching of the C-O-H group and two bands at 1079 and 1020 cm^-1^ are assigned to C-O bond stretching of the C-O-C group in the anhydroglucose ring of starch [29]. The absorptions at 1300–900 cm^-1^ might be allocated to the silicon compounds and silicates, which is ascribed to the Si-C or Si-O fundamental stretching vibration [30]. The silicon compound may coexist with other compositions in the rice due to the presence of rice husk.

**Figure 5:**
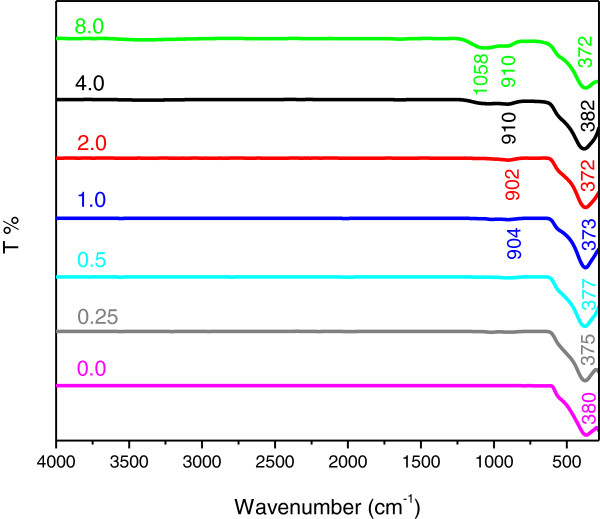
**FTIR spectra of ZnO samples synthesized at various concentrations of UR in the range of 4000–280 cm**^**-1**^** after calcination.**

Figure 5 shows the FTIR spectra for the ZnO nanostructures synthesized using various concentrations of UR after calcinations at 500°C for 5 hours. All the absorption bands due to rice disappeared from the FTIR spectra. Two new bands at about 900 cm^-1^ and 1058 cm^-1^ appear after calcinations treatment which can be assigned to the skeleton vibrational mode of glycosidic linkage (C-O-C) and C-H bending of starch molecules [31]. The characteristic bands of ZnO before calcination can be observed at about 350–390 cm^-1^ which after calcinations shifted to higher wavenumbers of 370–380 cm^-1^.

### Thermal analysis

**Figure 6:**
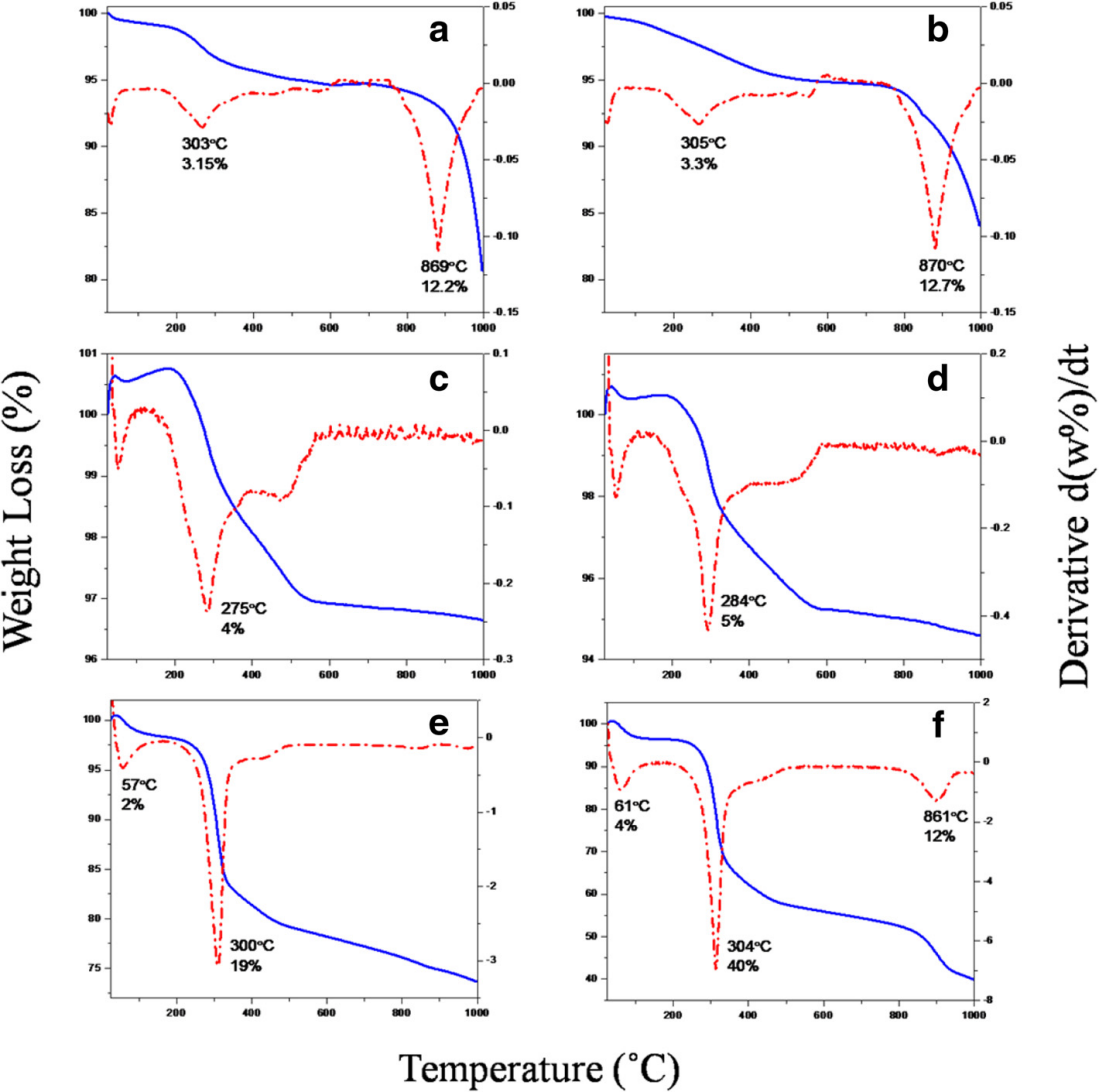
Thermogravimetric and differential thermogravimetric thermograms (TGA-DTG) of as-synthesized ZnO samples synthesized using various concentrations of UR (a) 0.25, (b) 0.5, (c) 1, (d) 2, (e) 4 and (f) 8 g.

Figure 6 shows thermal analysis of the as-synthesized ZnO synthesized using various concentrations of UR (0.25, 0.5, 1, 2, 4 and 8 g). Generally, three main decomposition steps can be observed; the first weight loss occurs at below 100°C, assigned to dehydration of water. The percentages of water loss increased from low concentration of rice (0.25 g) with below 1% weight loss to the highest one (8 g) with 4% weight loss. The low values of water loss are not shown in the thermograms. The second weight loss at about 300°C is assigned to the decomposition of rice flour components, mainly carbohydrates [32]. It is noteworthy that similar to the first decomposition, the weight loss percentages for second step increased with the increasing of the rice concentration in the samples. In other words, weight losses of 3.15, 3.3, 4, 5, 19 and 40% can be observed for samples with 0.25, 0.5, 1, 2, 4 and 8 g UR, respectively. Similar results were also reported by Zhang et al. [20] indicating that weight loss in the range of 190–490°C is attributed to the thermal decomposition and oxidation of soluble starch. The third decomposition at above 850°C is due to the degradation of the rice components residual with zinc acetate [33].

### Surface properties

**Figure 7:**
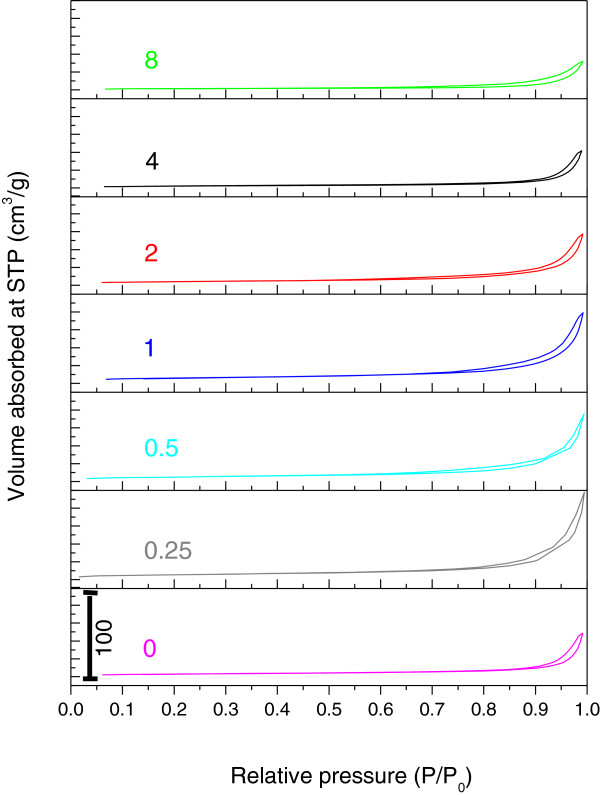
Nitrogen adsorption-desorption isotherms of as-obtained ZnO nanostructures synthesized at different concentrations of UR.

The nitrogen adsorption-desorption isotherms for ZnO nanoparticles prepared at different concentrations of UR are shown in Figure 7. All the isotherms can be ascribed as Type IV according to IUPAC classification, indicating mesopores-dominated property. Moreover, their hysteresis loop is interpreted as Type H3 representing aggregates of plate-like particles giving rise to slit-shaped pores. On the basis of the results from Figure 7, adsorption on these samples proceeds via multilayer formation in such a manner that the amount adsorbed increases gradually as the relative pressure increases. However, at higher pressures the amount adsorbed rises very steeply due to the capillary condensation in pores.

Generally, the volume absorbed for samples synthesized using 0.25, 0.5, 1 and 2 g UR is higher than that of ZnO synthesized without UR. It is notable that maximum volume absorbed is observed for the sample synthesized using 0.25 g UR with value of 98 cm^3^/g. The decreasing trend for volume absorbed value for rest of the samples can be clearly observed from Figure 7. These dramatic changes in the adsorption behavior are often caused by surface modifications, which manifested itself in a significant decrease in the amount adsorbed [34]. The desorption branches of the isotherms are also in good agreement with this result. The desorption branches of samples are quite different due to the modification has occurred in their pore’s texture.

In addition, H3 hysteresis usually indicates loosely assembled aggregated plate-like particles forming slit-like pores. However, changes can be observed from hysteresis loops. In the case of the sample synthesized using 8 g UR, an almost horizontal desorption branch with least volume adsorbed can be observed. Therefore this shows that modification in the pore texture of ZnO samples synthesized at different concentrations of UR has been occurred.

**Figure 8:**
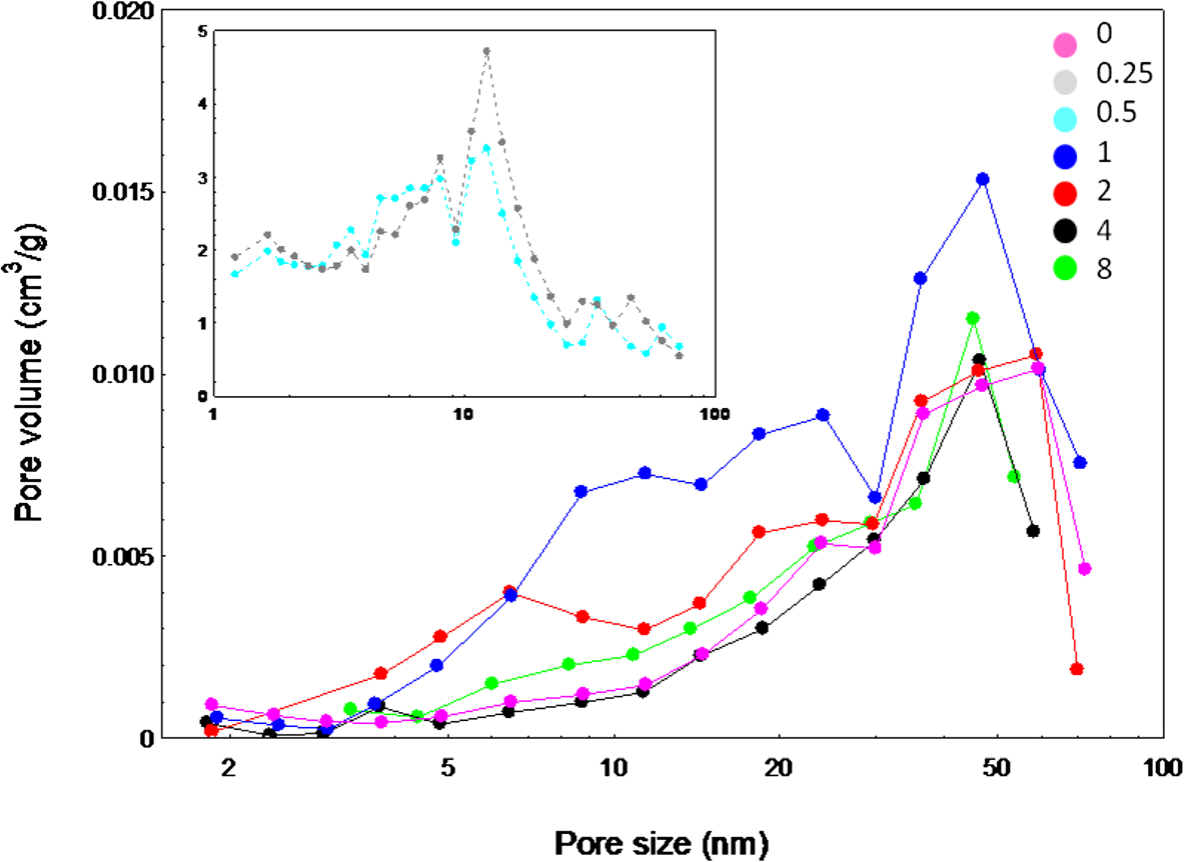
Barret-Joyner-Halenda (BJH) pore size distribution of ZnO nanostructures synthesized at different amounts of UR.

Figure 8 shows the Barret-Joyner-Halenda (BJH) pore size distribution for the samples synthesized at different concentrations of UR. As clearly seen from the plots, pores are located mainly between 2–50 nm, indicating that the material is dominated by mesoporous structure, which is in good agreement with the results of Type IV adsorption-desorption isotherms. However, due to the observation of hysteresis loops which shifted to a high relative pressure of P/P_0_ ≈ 1, the presence of large pores (>50 nm) is possible [35]. For better comparison, pore size distribution of as-synthesized ZnO synthesized without UR is also given, showing rather similar property to that of ZnO synthesized using 2 g UR with size distribution of around 60 nm with more than one distribution. The pore sizes of ZnO samples prepared using 0.25, 0.5, 1, 4 and 8 g UR are distributed at around 12, 12, 48, 47 and 46 nm, respectively. Due to known non-porous structure of ZnO, the pores observed were due to inter- and intra-particles agglomeration of secondary and tertiary types.

**Figure 9:**
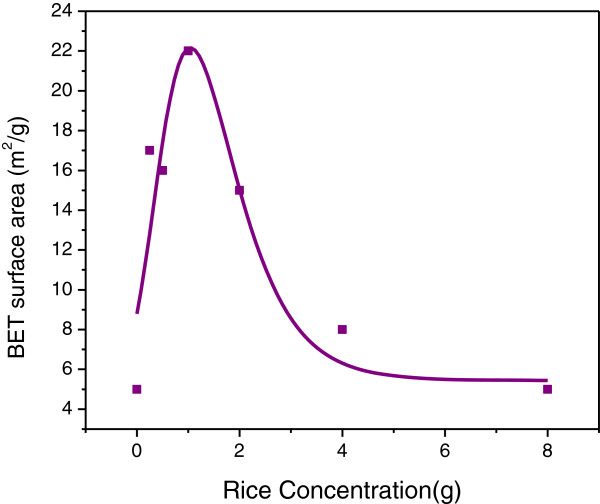
Plot of BET surface area against amount of UR for ZnO nano- microstructures synthesized using various concentrations of UR; 0, 0.25, 0.5, 1, 2, 4 and 8 g.

The BET surface area and the average pore diameter and pore volume of ZnO nanostructures synthesized at various concentrations of UR are listed in Table 1. The average pore diameter of the samples lies between 20–28 nm. The maximum pore volumes for samples synthesized using 0.25 and 1 g UR are the same, 0.12 cm^3^/g. BET surface area values for ZnO nanostructures synthesized at different concentrations of UR is shown in Figure 9. BET surface area value increase with the increasing of the uncooked rice amount up to 22 m^2^/g (at 1 g UR) and then decreased thereafter. The decrease in surface area is presumably due to the formation of bigger agglomeration at high UR ratio more than 1 g. The optimum BET surface area was observed for the ZnO sample synthesized using 1 g UR.

**Table 1:** BET surface area, BJH pore size diameter and pore volume of ZnO nano- microstructures synthesized at different concentrations of UR

**UR (g)**	**BET surface area (m^2^/g)**	**BJH pore diameter (nm)**	**BJH pore volume (cm^3^/g)**
0.0	5	23	0.07
0.25	17	28	0.12
0.5	16	26	0.10
1.0	22	20	0.12
2.0	15	20	0.08
4.0	8	25	0.06
8.0	5	20	0.04

### Optical properties

**Figure 10:**
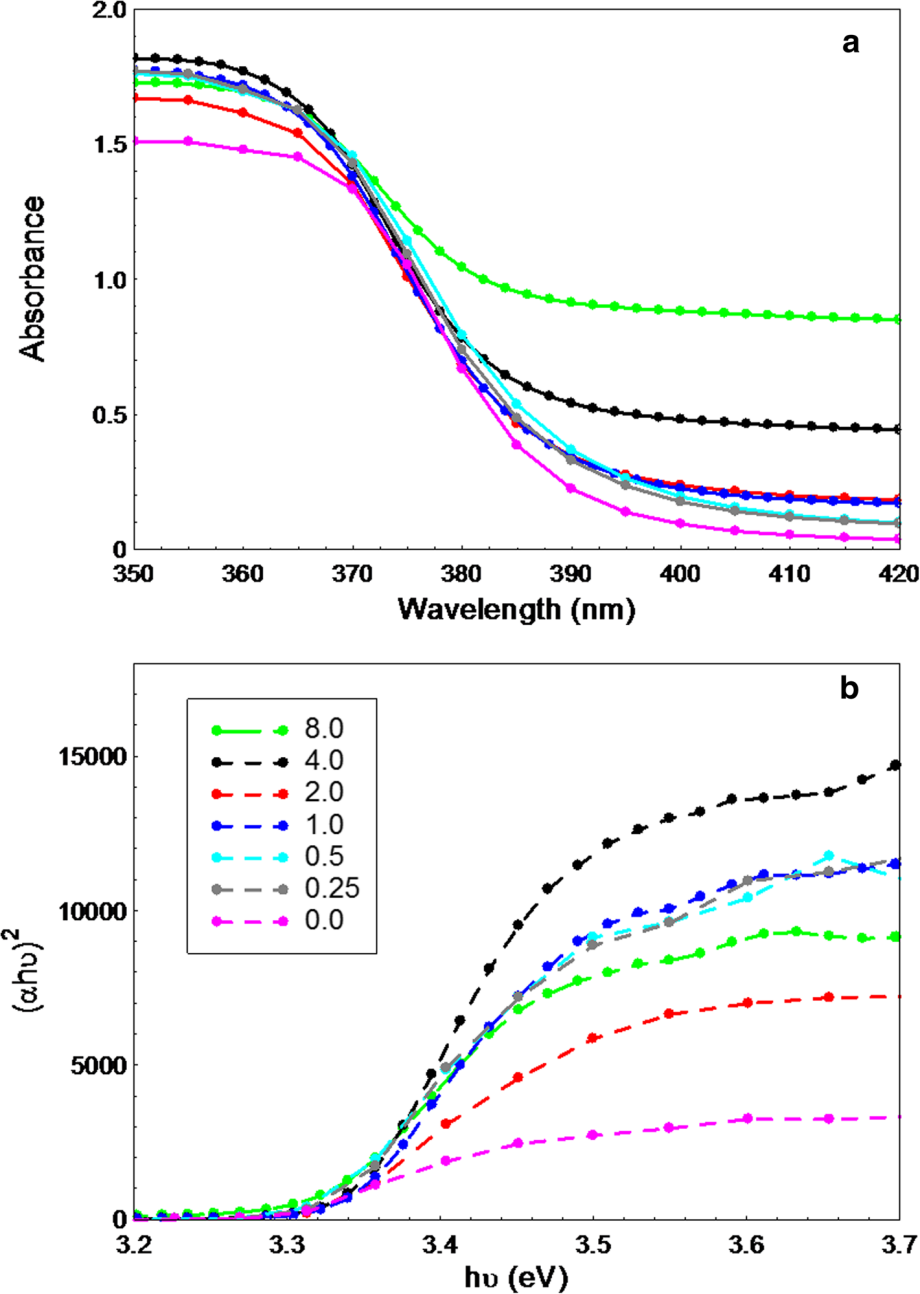
UV-visible absorption spectra (a) and Band gap energy (b) of as-synthesized ZnO nanostructures synthesized at different concentrations of UR.

The UV-visible absorption spectra of as-synthesized ZnO nanostructures are shown in Figure 10a. All the curves show absorption below 400 nm, corresponding to the intrinsic band gap of ZnO which is related to electron transitions from the valence band to conduction band. In addition, the samples synthesized at different concentrations of UR indicate higher UV–vis absorption compared to the one synthesized without UR. The direct-band gap energies as shown in Figure 10b were estimated from the plots of the transformed Kubelka-Munk function (αhυ)^2^ versus the photon energy (hυ). As seen in Figure 10b, linear region of the plot can be extrapolated to intersect the x-axis, and this value is identified as E_g_, the band gap energy. The E_g_ of as-synthesized ZnO prepared using 0, 0.25, 0.5, 1, 2, 4 and 8 g UR were found to be very similar, 3.29, 3.32, 3.32, 3.34, 3.32, 3.34 and 3.32 eV, respectively, in agreement with similar crystal structure as indicated by XRD results.

## Experimental procedure

All chemicals used in this work were of analytical reagent grade and used as received without any further purification. All aqueous solutions were prepared using deionized water. The raw rice was purchased from a local market and then ground into powder form in a milling machine, Fritsch Pulverisette 6 type planetary monomill, Germany.

In a typical procedure, 1 g of zinc acetate (Zn(Ac)_2_.2H_2_O) and 0.8 g sodium hydroxide (NaOH) were dissolved in 25 mL distilled water under constant stirring (Zn^2+^:OH^-^ = 1: 4). The measured pH was 13. After 1 hour stirring, different concentrations of rice powder 0, 0.25, 0.5, 1, 2, 4 and 8 g were introduced into the solution (the ratio of zinc acetate to rice powder was chosen at i.e. 1:0, 1:0.25, 1:0.5, 1:1, 1:2, 1:4 and 1:8 w/w%) and stirring was continued until the rice powder was completely dissolved. The solutions with lower concentrations of rice powder were easily dissolved and the color of solution remained white. In higher concentrations of rice powder, yellowish solutions after longer time of stirring were observed. Attempt has been made to use higher concentrations of rice, 16 g, but the resulting solution was very difficult of dissolve and therefore the experiment was discarded. Finally the mentioned solution was transferred into a Teflon-lined stainless steel autoclave, 50 mL and hydrothermal growth was carried out at 120°C for 18 h. After treatment, the autoclaves were allowed to cool down and the precipitates were collected, centrifuged at 40,000 × g for 10 min and supernatant was discarded. The obtained particles were washed three times with ethanol and distilled water in order to remove impurities and dried at 60°C for 24 h.

## Characterization

Powder X-ray diffraction (PXRD) analysis was performed on a Shimadzu diffractometer, XRD-6000 (Tokyo, Japan) equipped with CuK_α_ radiation. The morphology of the micro- and nanostructures were characterized by field emission scanning electron microscopy (FESEM) a JOEL JSM-6400 (Tokyo, Japan). Surface characterization of the material was carried out using nitrogen gas adsorption–desorption technique at 77 K using a Micromeritics ASAP 2000 (Norcross, GA, USA). Thermogravimetric and differential thermogravimetric analysis (TGA-DTG) were carried out using a Mettler Toledo instrument (Greifensee, Switzerland) using a heating rate of 10°C/min, in the range of 25–1000°C under nitrogen atmosphere. Fourier transform infrared spectra were recorded over the 280–4000 cm^−1^ range using a Perkin-Elmer 100 spectrophotometer (Waltham, MA, USA) under standard conditions. The UV-VIS-NIR spectrophotometer UV-3600 SHIMADZU was used to determine the optical properties.

## Conclusion

Uncooked rice, a bioresource can be used as biotemplate for morphology directing agent for the synthesis of ZnO micro- and nanostructures by hydrothermal method. The effects of uncooked rice on ZnO properties were investigated. Various ratios of rice were used and was found to change the morphology and size of the resulting ZnO crystals with different structures; flower-, flake-, rose-, star- and rod-like. The growth mechanism of ZnO crystals is possibly directed by conjugated and/or competing chemical/supramolecular interaction between zinc ions and the main component of the rice, carbohydrates. Surface modification of rice on the resulting ZnO was observed through pore texture and specific surface area.

## Abbreviations

BET: Brunauer-Emmett-Teller; BJH: Barret-Joyner-Halenda; DTG: Differential thermogravimetric analysis; Eg: Band gap energy; eV: Electron volt; FESEM: Field emission scanning electron microscopy; FTIR: Fourier transform infrared; g: Gram; h: Hour; IUPAC: International union of pure and applied chemistry; JCPDS: Joint committee on powder diffraction standards; nm: Nanometer; No.: Number; TGA: Thermogravimetric analysis; UR: Uncooked rice; UV: Ultraviolet; XRD: X-ray diffraction; Zn2+: Zinc cation; ZnO: Zinc oxide.

## Competing interests

There is no conflict of interest for all authors of this article.

## Authors’ contributions

DR is the first author of this article. MZBH is the second and correspond author. YHTY is the third author. All authors read and approved the final manuscript.
